# Estimation of daily interfractional larynx residual setup error after isocentric alignment for head and neck radiotherapy: quality assurance implications for target volume and organs‐at‐risk margination using daily CT on‐rails imaging

**DOI:** 10.1120/jacmp.v16i1.5108

**Published:** 2014-01-08

**Authors:** Charles A. Baron, Musaddiq J. Awan, Abdallah S.R. Mohamed, Imad Akel, David I. Rosenthal, G. Brandon Gunn, Adam S. Garden, Brandon A. Dyer, Laurence Court, Parag R. Sevak, Esengul Kocak‐Uzel, Clifton D. Fuller

**Affiliations:** ^1^ Department of Radiation Oncology The University of Texas M. D. Anderson Cancer Center Houston TX USA; ^2^ Jefferson Medical College Philadelphia PA USA; ^3^ Department of Radiation Oncology Case Western Reserve University Cleveland OH USA; ^4^ Department of Clinical Oncology Faculty of Medicine Alexandria University Alexandria Egypt; ^5^ American University of Beirut Medical Center Beirut Lebanon; ^6^ Department of Radiation Oncology Şişli Etfal Teaching and Research Hospital Istanbul Turkey; ^7^ Department of Radiation Medicine Oregon Health & Science University Portland OR USA; ^8^ Graduate School of Biomedical Science University of Texas Health Science Center Houston TX USA; ^9^ University of Texas Medical Branch Galveston TX USA

**Keywords:** image‐guided radiotherapy, head and neck radiotherapy, interfractional larynx setup error, quality assurance, target volume and organs‐at‐risk margins

## Abstract

Larynx may alternatively serve as a target or organs at risk (OAR) in head and neck cancer (HNC) image‐guided radiotherapy (IGRT). The objective of this study was to estimate IGRT parameters required for larynx positional error independent of isocentric alignment and suggest population‐based compensatory margins. Ten HNC patients receiving radiotherapy (RT) with daily CT on‐rails imaging were assessed. Seven landmark points were placed on each daily scan. Taking the most superior‐anterior point of the C5 vertebra as a reference isocenter for each scan, residual displacement vectors to the other six points were calculated postisocentric alignment. Subsequently, using the first scan as a reference, the magnitude of vector differences for all six points for all scans over the course of treatment was calculated. Residual systematic and random error and the necessary compensatory CTV‐to‐PTV and OAR‐to‐PRV margins were calculated, using both observational cohort data and a bootstrap‐resampled population estimator. The grand mean displacements for all anatomical points was 5.07 mm, with mean systematic error of 1.1 mm and mean random setup error of 2.63 mm, while bootstrapped POIs grand mean displacement was 5.09 mm, with mean systematic error of 1.23 mm and mean random setup error of 2.61 mm. Required margin for CTV‐PTV expansion was 4.6 mm for all cohort points, while the bootstrap estimator of the equivalent margin was 4.9 mm. The calculated OAR‐to‐PRV expansion for the observed residual setup error was 2.7 mm and bootstrap estimated expansion of 2.9 mm. We conclude that the interfractional larynx setup error is a significant source of RT setup/delivery error in HNC, both when the larynx is considered as a CTV or OAR. We estimate the need for a uniform expansion of 5 mm to compensate for setup error if the larynx is a target, or 3 mm if the larynx is an OAR, when using a nonlaryngeal bony isocenter.

PACS numbers: 87.55.D‐, 87.55.Qr

## I. INTRODUCTION

While intensity‐modulated radiotherapy (IMRT) has led to the ability to deliver highly conformal radiotherapy (RT) doses, a major limitation in sparing normal tissues while delivering tumoricidal doses to target volumes, after target delineation, is setup error.^1^, ^2^ Conceptually, in ICRU62^(3)^ and ICRU 83,^(4)^ the planning target volume (PTV) and planning organs‐at‐risk volume (PRV) account for this setup error and ensure that precision target delineation does not result in either a geometric miss nor inadvertent normal tissue overdose.^5^, ^6^ However, a significant limitation of most current image‐guided radiation therapy (IGRT) systems is their reliance on a single point reference for corrective setup translations. For example, use of a single isocenter for portal imaging or an index slice or contour for cone‐beam CT data^(7)^ has consequences in the head and neck, where target structures or organs at risk (OARs) are not necessarily fixed to bony landmarks and experience translational motion during delivery of radiation.^8^, ^9^, ^10^ Consequently, despite excellent setup, TV/OAR displacement from the isocenter may occur in a directionally distinct manner.^(11)^ Recently, there has been increased interest in efforts to spare the carotid arteries from significant dose for early‐stage laryngeal cancer.^12^, ^13^, ^14^ To this end, IMRT is therapeutically justified, owing to the fact that these cancers are, by and large, highly curable^(15)^ and long‐term toxicity, therefore, becomes a significant consideration in patients with potential decades of survival. Additionally, our group and others have adopted strategies to minimize laryngeal doses for nonlaryngeal head and neck cancers when a low neck match cannot be utilized practically.^(16)^ Such approaches are beneficial for organs like larynx where a defined planning organ at risk volume (PRV) margin might be of possible value for plan optimization as it is well documented that laryngeal overdose results in quantifiable toxicity.^(17)^

While masks can serve to reduce patient external motion during treatment, internal target/organ movement is an unavoidable reality that must be considered, as well, for treatment accuracy. However, it is imperative that the use of IMRT does not result in inadvertent geometric miss which may in aggregate reduce survival probability. For this reason, we sought to ascertain the relative geometric variation in the motion of the larynx relative to a single isocenter (defined as a bony landmark) in order to ensure that our current radiotherapy margins are within evidence‐based limits.

The specific aims of the current study are:

1) estimation of the relative interfraction setup error of the laryngeal apparatus relative to a fixed isocenter, using both experimentally observed CT on‐rails data and robust estimators of population setup error using a bootstrap methodology;
2) determination of the PTV expansions required for laryngeal‐targeting radiotherapy for larynx cancers; and
3) estimation of PRV expansions required for laryngeal sparing‐radiotherapy for nonlaryngeal head and neck cancers

## II. MATERIALS AND METHODS

**Figure 1:**
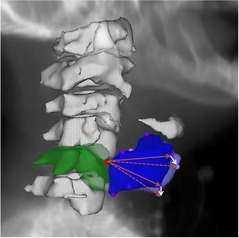
Visual depiction of the selected points of interest (POIs) with the red circle at the most superior aspect of the anterior C5 vertebra (3D reconstructed contour in green) representing the fixed isocenteric reference and the violet circles represent selected POIs of the thyroid cartilage on Day 1 (3D reconstructed contour in blue), while the pink circles represent the same POIs on Day 2 (not all POIs are visible because of the overlap) but in different spatial location caused by laryngeal inter‐fraction motion. White arrows show example of vector displacements of two POIs relative to their original position in relation to the fixed isocenter of Day 1 (solid red arrow) in Day two (dashed red arrow).

Daily DICOM RT from a series of ten patients previously enrolled on an adaptive RT study^18^, ^19^ were de‐archived after IRB approval. Daily noncontrast CT on‐rails (350 mm FOV, 1×1×2.5 mm voxel dimensions)^20^, ^21^ scans were acquired, as detailed previously,^18^, ^19^ and imported into a treatment planning system (Pinnacle; Philips Healthcare, Andover, MA). For each daily CT on‐rails DICOM 3D image, the C5 vertebra and thyroid cartilage were identified and seven reference points were manually placed as a point of interest (POI) at the superior‐most voxel of the anterior aspect of the C5 vertebrae, the superior‐most and inferior‐most voxels of the anterior aspect of thyroid cartilage, as well as the superior‐most and inferior‐most voxels of the most lateral aspect of the right and left thyroid cartilage cornua (see Fig. 1). The selection of larynx six POIs was based on the fact that thyroid cartilage is the largest laryngeal cartilage that forms the external framework of the larynx and houses its structural components with strong attachments. The selected landmark points shape a three‐dimensional framework of the upper‐ and lower‐most boundaries of the thyroid cartilage in the midline and bilaterally to best represent laryngeal motion.

The most superior–anterior point of the C5 vertebra was defined a priori as a fixed origin for each daily scan. On each daily CT on‐rails scan, vector displacements were obtained to the other six reference POIs relative to this origin. All such vectors were brought to the same origin and, as such, the vectors represent the motion of the larynx relative to this origin. Conversely, the larynx represented by the six‐point structure, represents a three‐dimensional registered object moving relative to a fixed point. This isolates laryngeal motion changes relative to a point in the bony anatomy independent of errors in patient setup. The geometry is the same in every CT and any setup changes would result in only translational or rotational shifts, which would be negated by the use of vectors. Assuming each patient's initial verification (Day 1) scan was treated as a “gold standard” reference, vector differences between the initial verification scan and each daily CT on‐rails scan was calculated serially. The magnitudes of these vector differences were collected, representing the daily shifts of the entire larynx relative to a fixed point from a planned setup, and as a measure of the intrinsic movement of the larynx (i.e., the motion of the larynx despite immobilization and changes in day‐to‐day controlled setup) (see Fig. 1).

The mean magnitude of vector displacement over all days was calculated for each patient at each POI. From these mean values, a grand means was calculated at each point to characterize the cumulative displacement for each POI. The systematic error for the population was defined as the standard deviation of the grand mean. The random error for each individual was determined to be the standard deviation about an individual's mean value for each vector, while the population random error is given by the root mean square of individual random errors (see Fig. A1). The results from these calculations, the observational cohort systematic error ($\Sigma_{\text{cohort}}$) and the random error ($\sigma_{\text{cohort}}$), were used to calculate the necessary CTV‐to‐PTV correction sufficient for 90% of patients to receive 95% of the nominal dose for each POI, using Van Herk's formula:^(22)^

(1) CTV‐to‐PTV margin=2.5∑+0.7σ

Additionally, using this data, the necessary PRV is calculated based on work by McKenzie et al.^(23)^ and is calculated as:

(2) OAR‐to‐PRV margin=1.3∑+0.5σ

To calculate robust nonparametric estimates for inference, bootstrap resampling was applied using Efron's bootstrap methodology. Using an iterative resampling/replacement method, a cumulative 1,000 random distributions for each POI, was drawn from each individual patient's distribution of daily shifts using the original 1854 individual experimentally derived daily POI displacement measures. The resultant $6\times 10^{5}$ resampled distributions (i.e., 6 POIs×10 original patient distributions×10,000 replacement/resampling iterations) were then used to generate a robust systematic error ($\Sigma_{\text{bootstrap}}$) and random error ($\sigma_{\text{bootstrap}}$) for robust probabilistic estimation of the population‐level magnitude of larynx interfractional motion at each POI. Likewise, a 95% tolerance interval (i.e., a 95% confidence interval of a range encompassing 95% of all displacements) was derived as an estimator of the internal target volume for both cohort and bootstrap distributions (95% ${\text{TI}}_{\text{cohort}}$ and 95% ${\text{TI}}_{\text{bootstrap}}$).

## III. RESULTS

**Figure 2:**
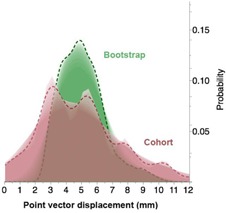
Shadowgram showing the difference in distribution probability of points of interest vector displacement over treatment time between the studied cohort and its bootstrap resampling.

A total of 309 daily CT on‐rails DICOM images were utilized for all ten patients, an average of 31 scans per patient, with a minimum of 25 scans and a maximum of 35 daily scans. Mean observational cohort (n=10 patients) displacement for each anatomic POI ranged from 4.77 to 5.30 mm, with a grand mean of 5.07 mm over‐and‐above the correction for bony landmark setup error among all points. Calculated systematic error $\Sigma_{\text{cohort}}$ for all POIs ranged between 1.01–1.38 mm, with a mean $\Sigma_{\text{cohort}}$ of 1.1 mm across all points, and random error $\sigma_{\text{cohort}}$ of 2.48–2.87 mm with a mean $\sigma_{\text{cohort}}$ of 2.63 mm across all points. For all sites, the bootstrapped POI mean displacement ranged from 4.85–5.35 mm, with a grand bootstrapped POI mean displacement of 5.09 mm. Figure 2 illustrates the difference in POI vector displacement distribution probability between the studied cohort and its bootstrap resampling.

Calculated $\Sigma_{\text{bootstrap}}$ for all POIs ranged between 1.06–1.46 mm, with a mean $\Sigma_{\text{bootstrap}}$ of 1.23 mm across all resampled points, and $\sigma_{\text{bootstrap}}$ of 2.45–2.85 mm with a mean $\sigma_{\text{bootstrap}}$ of 2.61 mm (Table 1).

**Figure 3:**
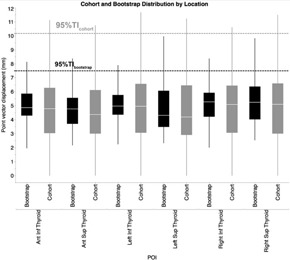
Distributional boxplot of geometric vector displacement of cohort POIs and its bootstrap validation. Pale line within the box indicates median value, while the box limits indicat the 25th and 75th percentiles. The lines represent the 10th and 90th percentiles, and the horizontal dotted lines represent the 95% TI.

For the observed patient cohort, the one‐sided upper limit ensuring 95% coverage of all residual displacements ($95\%\;{\text{TI}}_{\text{cohort}}$) was 10.18 mm, while the equivalent bootstrap‐estimated population limit ($95\%\;{\text{TI}}_{\text{bootstrap}}$) dropped to 7.52 mm (Fig. 3).

Using van Herk's formula ^(22)^ (Eq. (1)) for the observed cohort, the margin required for CTV‐ to‐PTV expansion to ensure 90% population coverage with 95% of prescribed dose (${\text{PTV}}_{\text{cohort}}$) was 4.6 mm over‐and‐above the correction for bony landmark setup error, while the calculated bootstrap estimator of the equivalent requisite coverage margin (${\text{PTV}}_{\text{bootstrap}}$) was 4.9 mm. Moreover, using McKenzie formula^(23)^ (Eq. (2)), the calculated OAR‐to‐PRV expansion for the observed residual setup error (${\text{PRV}}_{\text{cohort}}$) was 2.7 mm, closely approximating the bootstrap estimated expansion over all POIs (${\text{PRV}}_{\text{bootstrap}}$) of 2.9 mm.

**Table 1:** Statistical data corresponding to individual POIs for the studied cohort and the bootstrap validation

*Point*	*Mean, Cohort Vector Displacement (mm)*	*Mean, Bootstrapped Vector Displacement (mm)*	Σ*, Cohort Systematic Error (mm)*	σ*, Cohort Random Error (mm)*	Σ*, Bootstrapped Systematic Error (mm)*	σ*, Bootstrapped Random Error (mm)*
Most Superior Aspect of the Anterior Thyroid Cartilage	4.77	4.85	1.17	2.59	1.24	2.57
Most Inferior Aspect of Anterior Thyroid Cartilage	4.97	5.01	1.01	2.65	1.06	2.62
Most Superior Aspect of Left Superior Cornu of Left Thyroid Cartilage	5.16	5.10	1.1	2.48	1.20	2.46
Most Inferior Aspect of Left Inferior Cornu of Thyroid Cartilage	5.09	5.10	1.23	2.48	1.30	2.45
Most Superior Aspect of Right Superior Cornu of Thyroid Cartilage	5.3	5.35	1.38	2.87	1.46	2.85
Most Inferior Aspect of Right Inferior Cornu of Thyroid Cartilage	5.08	5.13	1.07	2.73	1.13	2.69

## IV. DISCUSSION

The goal of in‐room, image‐guided radiotherapy (IGRT) is to improve treatment delivery via a reduction of PTV volumes by imaging patients before or during treatment. Consequently, high‐frequency head and neck IGRT may be used as a feedback mechanism, ensuring accuracy of patient setup and providing an opportunity to adjust the PTV^24^, ^25^ or PRV^(26)^ to account for institutionally dependent setup error.^27^, ^28^

In HNC, the accuracy of RT is of extreme importance owing to the close proximity of many OARs to the target volumes; the larynx, as either a target (PTV) or avoidance (PRV) structure is no exception. Our efforts herein define evidence‐based, institutional margins for cases when the laryngeal apparatus is a target (i.e., larynx cancer)^12^, ^13^, ^14^, ^29^ and define reasonable preplanning PRV margins to avoid beam‐path toxicities when laryngeal sparing is desired^30^, ^31^ (i.e., oropharynx/oral cavity cancers receiving elective neck radiation when a low‐neck match approach is not feasible).^16^, ^32^

In HNC treatment, immobilization masks are used to minimize interfractional variation in patient setup and motion during radiation delivery. The amount of error in daily setup in an immobilized patient has been studied previously^33^, ^34^, ^35^ and CTV‐PTV corrections necessary have been suggested previously using a variety of IGRT devices.^7^, ^36^ Interestingly, while studies have been performed to determine the effect that setup error and the movement of a patient as a whole can have on RT accuracy in HNCs, there has been a lack of analysis of the effects of independent TV/OAR/ROI motion, although this data is beginning to emerge.^9^, ^21^, ^26^, ^27^, ^29^

In head and neck cancers, the larynx provides an ideal model for nonisocentric treatment planning and patient setup as it potentially has large translational displacements relative to bony landmarks due to its flexible attachments. Additionally, the cartilaginous structures of the larynx are capable, on some level, of deformation throughout treatment, thus necessitating the use of multiple points in the assessment of this study. As an illustration, when serial daily CT scans are concatenated in movie format (see Appendix A) the concomitant effect of bony and laryngeal setup error is better appreciated. With traditional patient setup and immobilization, the large, dynamic changes in the laryngeal apparatus, as seen in Appendix A, are not appreciated or accounted for with daily isocentric alignment to rigid structures. Furthermore, as highly conformal approaches become the standard of practice, IGRT implementation can further improve outcomes through the direct assessment of interfractional laryngeal variability and laryngeal motion during radiation delivery. For example, at our institution, the entire larynx is routinely treated in cases with locally advanced disease; evaluation of kilovoltage portal radiography allows alignment of laryngeal structures directly rather than solely relying on bony landmarks — a feat that would have proven difficult in the megavoltage portal imaging era. Additionally, volumetric approaches, including CT on rails or cone‐beam CT, may be utilized with similar purpose. When using the larynx in toto for positional alignment, the proposed margins may be utilized directly for CTV‐PTV expansion, whereas with a nonlarynx‐based reference (e.g., bony landmark), the PTV margins require superimposition for isocentric alignment of the reference structure. Our data show that, utilization of IMRT for early stage larynx cancers^(14)^ and emerging interest in even more aggressive and technical IGRT strategies, necessitate the need to mitigate intrafractional setup error^38^, ^39^, ^40^ and thus require excellent geometric accuracy throughout the setup and delivery of treatment. For instance, CT on rails has impressive isocentric alignment performance characteristics (reported by Shiu et al.^(41)^ with <0.5 mm directional error and <1 mm cumulative isocentric error when aligning to spinal structures).

In nonlaryngeal HNC, our data are equally important because OARs require dosimetric boundaries, as well, to ensure that overdosage does not occur. This is conceptually represented as a PRV.^(42)^ At our institution, a low‐neck match is used whenever feasible;^16^, ^32^, ^43^ however, alignment of the isocenter for the IMRT field must still account for potential laryngeal setup error during treatment in order to ensure that unanticipated dose overlap does not occur. Several authors have demonstrated that extraneous, but modifiable, laryngeal dose is associated with significant acute and long‐term toxicities.^44^, ^45^ Institutions using a full neck IMRT strategy would be prudent to consider PRV margination with magnitudes comparable to those listed, in order to ensure attempts at organ sparing are effectively realized.

Our data suggest that a population‐based CTV‐PTV margin of 5 mm reasonably accounts for larynx motion if the larynx is a target structure, or 3 mm if it is planned as a PRV before dose calculation (i.e., preoptimization), using established margination recipes.^22^, ^23^ These adjustments sufficiently account for geometric error associated of the laryngeal apparatus during setup and treatment execution, ensuring precise delivery of the prescribed dose for all measured values. Our study used a postisocentric alignment, suggesting that if a bony reference isocenter is used, an additional margin is required. However, if the laryngeal anatomy itself is used as alignment reference, the use of a bony (C5) isocenter may be obviated. Institutionally we now align directly to laryngeal structures.

There are several caveats inherent in our data. First, this series represents a limited number of patients from a single institution with serial imaging for predominately oropharyngeal cancers. Furthermore, our exclusive use of daily CT on rails does not allow for evaluation of intrafractional respiratory or swallowing motion associated with the larynx^40^, ^46^, ^47^ which may require additional margination, and which we and other groups are currently investigating. The use of bootstrap resampling serves as a corrective for our limited sample size, as the large number of iterative daily measurements (>25 for any patient, 1854 total measurements) and the large resampling run (n=10,000 per patient, per POI) allows robust inferential estimation, at least for similar populations. The bootstrap distribution (and resultant systematic, random error, and confidence intervals) are designed to represent a large‐scale population from which the sampled experimental set is potentially drawn. Consequently, as expected some differences exist (as the resampled population central parameters will be of more utility, as compared to experimental cohort data), but on the whole, the magnitude of difference between bootstrap and experimental cohort for systematic and random error was for all measures <0.2 mm (exceedingly small). This, in fact, suggests our presented experimental data largely would reflect any given head and neck cancer patient larynx motion distribution that might be seen in a significant patient cohort.

This is, however, to our knowledge, the first study to utilize diagnostic quality imaging via daily CT on rails to evaluate interfractional motion of the larynx. It serves as a model study to evaluate interfractional organ motion for delivery of IGRT, as well as a benchmark for institutional IGRT margination recipes.

## V. CONCLUSIONS

In conclusion, interfractional larynx setup error is a source of significant potential geometric error, even after pristine isocentric alignment, in HNC treated with radiation therapy, both when the larynx is treated as a target (e.g., larynx primaries) or as a normal tissue avoidance structure (e.g., oropharyngeal cancer). We estimate the need for a uniform CTV‐to‐PTV expansion of approximately 5 mm to compensate for daily isocenter‐independent setup error if the larynx is a target, or an OAR‐to‐PRV margin of 3 mm if the larynx is an OAR, when using a nonlaryngeal isocenter with comparable immobilization platforms.

## ACKNOWLEDGMENTS

Dr. Abdallah Mohamed acknowledges the support by a UICC American Cancer Society Beginning Investigators Fellowship funded by the American Cancer Society.

Dr. Fuller received/receives grant support from: the National Institutes of Health/National Cancer Institute's Paul Calabresi Clinical Oncology Award Program (K12 CA088084‐06) and Clinician Scientist Loan Repayment Program (L30 CA136381‐02); the National Institutes of Health Cancer Center Support (Core) Grant CA016672 to The University of Texas MD Anderson Cancer Center; the SWOG/Hope Foundation Dr. Charles A. Coltman, Jr., Fellowship in Clinical Trials; a General Electric Healthcare/MD Anderson Center for Advanced Biomedical Imaging In‐Kind Award: an Elekta AB/MD Anderson Department of Radiation Oncology Seed Grant; the Center for Radiation Oncology Research at MD Anderson Cancer Center. These listed funders/supporters played no role in the study design, collection, analysis, interpretation of data, manuscript writing, or decision to submit the report for publication.

## APPENDICES

### Appendix A: Dynamic daily changes in the laryngeal apparatus position after isocentric alignment

(view the movie, uploaded to www.jacmp.org as Supplementary Material).

**Figure Fig. A1.**
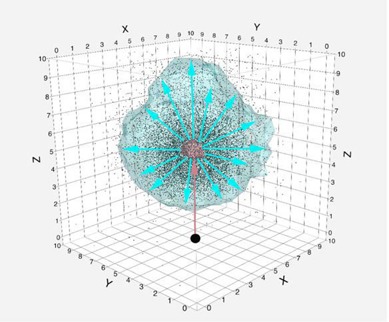
Three‐dimensional scatterplot illustrating the difference between the systemic errors of representative points of interest distribution from a starting point (black circle) presented as a red cloud and the random errors represented as a light blue cloud. The appropriate CTV‐PTV and OAR‐PRV margins accounts for both the systematic and random error component.

## Supporting information

Supplementary Material
